# Effect of menstrual irregularity on academic performance of undergraduate students of Debre Berhan University: A comparative cross sectional study

**DOI:** 10.1371/journal.pone.0280356

**Published:** 2023-01-26

**Authors:** Enguday Demeke, Abayneh Birlie Zeru, Esubalew Tesfahun, Woineshet Bedru Mohammed

**Affiliations:** 1 Department of Public Health, Debre Berhan University, Addis Abeba, Ethiopia; 2 Public Health, Director Medicine and Health Science Institute, Debre Berhan University, Addis Abeba, Ethiopia; University of Belgrade Faculty of Organisational Sciences: Univerzitet u Beogradu Fakultet organizacionih nauka, SERBIA

## Abstract

**Background:**

Menstrual period is a critical time in the life of females. For the first few years after menarche, irregular cycle is common due to premature hypothalamic-pituitary ovarian (HPO) axis physiologically. If persistent, it becomes a major problem in student’s life. But few studies conducted on effect of menstrual cycle irregularity on academic performance among university students were descriptive. However assessing the effect of menstrual irregularity on academic performance by using average grade point approach is critically important to magnify its effect.

**Objective:**

To compare effects of menstrual irregularity on academic performance among undergraduate students of Debre Berhan University, Ethiopia 2020.

**Methods:**

A comparative cross sectional study was conducted among 404 students in Debre Berhan University, Ethiopia, 2020. A standard tool of menstrual cycle regularity which is prepared by international federation of gynecologist and obstetrics (IFGO) was used to screen students who have menstrual irregularity. Independent sample t- test was done to compare the mean difference of academic performance between the two groups of students.

**Results:**

The age of study participants ranges between 18 and 26 years with a mean age of 20.69 ± 1.43 years. The mean age at menarche was 14.9 ± 1.67 ranging from 9 to 18 years. Students who had menstrual irregularity had mean average grade point (AGP) of (2.78 ± 0.57) and students who had regular menstruation had mean AGP of (2.97 ± 0.53). Students who had menstrual irregularity had lower mean average grade point by a mean difference of 0.19 (95%CI: 0.09–0.30).

**Conclusion:**

This study found that students who had menstrual irregularity had significantly lower mean AGP as compared with students who had regular menstruation.

## Introduction

Regular menstrual cycle (counting from the first day of one menstrual period to the first day of the next cycle) is 21 to 35 days and lasts from 3 to 7 days duration with volume of blood loss 5–80 ml [1]. Menstrual irregularity refer to any kind of changes occurring in regularity of onset, frequency of onset, duration of flow and volume from regular menstrual cycle [2–4].

Menstrual period has a notable effect on the academic performance of female students [5]. It influences social life, diet, exercise, amount of sleep, sleep quality, study time, concentration, group activities, preparation and performance on exam and attendance [6, 7]. Different studies showed that academic performance of women varies during their menstrual cycle, in a way that the mental status is decreased during and several days before the period [8, 9]. In addition, another study conducted in Saudi Arabia revealed that there was an increase rate of absenteeism and loss of concentration in academic work which might have impact on school performances as well as the achievement of their life goals [10]. Study conducted in Mansoura University revealed that more than two third of nursing students had sleeping desire during lectures [11].

The majority of researches regarding effect of menstrual irregularity have been focused on daily activities and descriptive. However assessing the effect of menstrual irregularity on academic performance by using average grade point approach is critically important. Therefore, the aim of this study was to compare the effect of menstrual irregularity on academic performance by using average grade result of the last semester among Debre Berhan university students.

## Methods

### Study design, settings and participants

Institutional based comparative cross sectional study design was conducted in Debre Berhan University from February 11 to March 10 /2020. Debre Berhan is located at 130 Kms from Addis Ababa (capital city of Ethiopia) and at 695 kms from Bahir Dar (the capital city of Amhara Region). There were about 5387 female students during the study period. Undergraduate regular students who were pregnant, who were within one year after delivery and lactating, who had treatment history for menstrual irregularity during the year of study and who were critically ill during data collection period were excluded from the study.

### Sample size and sampling procedures

The sample size was calculated using a formula for estimation of double population proportion with the assumption of small to medium effect sizes (Cohen’s d = 0.05), 95% confidence interval and a power of 80%. 202 students who have irregular menstrual cycle and 202 students who had regular menstrual cycle were included in the study. The study subjects were selected using simple random sampling (lottery method) technique from the list of the students.

### Data collection tools and procedures

A pretested self-administered questioner was used to collect the data. The questionnaire includes socio demographic questions, menestrual cycle pattern related questions, menstrual related symptoms questions, problems faced during menses questions and average grade point of the last semester taken from the students register. The questionnaire was first prepared in English, and translated into Amharic language. A person who was expert in both languages checked the questionnaires’ consistency. Two data collectors (graduated public health) and one supervisor (master of public health student) was participated throughout the data collection process.

### Measures

#### Outcome

The investigators want to compare the average grade point of students who have menstrual cycle irregularity with students who have menstrual cycle regularity. In order to assure the comparison similar in academic year only the last one semester average grade point was taken for all students who have regular and irregular menstrual cycle.

#### Predictors

Whether menstrual cycle is regular or irregular should be determined by using standards of menstrual irregularity definition which was prepared by international federation of gynecology and obstetrics (IFGO) 2018. Therefore, in the present study regular menstrual cycle was defined as if frequency of menses is 24–38 days, duration of bleeding less than or equal to 8 days, cycle to cycle variation over the last one year be less than 10 days and if the individual perception on amount is normal [12]. On the other hand menstrual irregularity refers to anything outside regular menstrual cycle limit.

### Data analysis

Epi-data version 3.1 was used for data entry and exported to SPSS version 21 software for analysis. Normality test (by using Kolmogorov-Simonov and Shapiro-wilk test), homogeneity of variance (by using Levene’s test) were done. Out layer detection was done by using box and whisker plot. Descriptive statistics such as frequency and percentage were computed for categorical variables. Continuous variables were presented as mean ± standard deviation or median. Independent sample t- test was done to compare the mean difference of academic performance between the two groups of students.

## Results

### Socio-demographic characteristics

The average age of the students was 20.69 ± 1.43 years old with a range of 18–26. Half of respondents 203 (50.20%) came from rural area and 346 (85.60%) of study participants were orthodox Christian followers (Table 1).

**Table 1:** Socio-demographic characteristics of undergraduate female students of DBU, Ethiopia participated in the study, 2020 (N = 404).

Variables		Frequency	Percent %
Age	18–20	209	51.73
	21–22	179	44.31
	23–26	16	3.96
Ethnicity	Amhara	298	73.76
	Oromo	68	16.83
	SNNP	16	3.96
	Tigrie	17	4.21
	Others	5	1.24
Residence before university admission	Urban	201	49.75
	Rural	203	50.25
Religion	Orthodox	346	85.64
	Protestant	30	7.43
	Muslim	27	6.68
	Others	1	0.25
Marital status	Single	376	93.07
	Married	26	6.44
	Others	2	0.50
Birr sent from family per month	<3USD	88	21.78
	3-6USD	116	28.71
	≥6USD	200	49.50

### Menstruation and menstrual irregularity

The mean menarcheal age of study subjects was 14.90 ± 1.67 ranging from 9 to 18 years. The average duration of the menstrual cycle lasted between 2–12 days with a mean of 4.84 ± 1.81 days.

Among menstrual irregularities, (those who have menstrual cycle length <24 days, menstrual cycle length > 38 days, inter menstrual difference > 10 days, perception on menstrual blood flow light, perception on menstrual blood flow heavy and menstrual blood flow duration > 8 days), irregular onset was the major problem 123 (30.45%). Light menstrual flow, heavy menstrual flow, prolonged period, frequent period and infrequent period accounts in 116 (28.71%), 80 (19.80%), 24 (5.94%), 23 (5.69%) and 3 (0.74%), respectively (Table 2).

**Table 2:** Pattern of menstrual cycle among undergraduate students of DBU, Ethiopia, participated in the study, 2020 (*N =* 404).

Variables		Frequency	Percent (%)
Length of menstrual cycle	<24 days	23	5.69
	24–38 days	204	50.50
	>38 days	3	0.74
Regularity of onset / Inter menstrual difference	Regular (<10 days)	51	12.62
	Irregular (*≥ 10 days)*	123	30.45
Menstrual blood flow duration	≤8 days	380	94.10
	>8 days	24	5.94
Perception on menstrual blood flow	Light	116	28.71
	Normal	208	51.49
	Heavy	80	19.80
Over all menstrual cycle	Irregular	202	50
	Regular	202	50

### Menstrual related symptoms

The majority experienced symptoms during their menstrual period were abdominal cramps 285 (70.54%), back pain 139 (34.41%), disappointment 94(23.27%) (Table 3).

**Table 3:** Menstrual related symptoms occur during menses among undergraduate students of DBU, Ethiopia, participated in the study, 2020 (N = 404).

Symptom	Number	Percentage (%)
abdominal cramps	285	70.54
back pain	139	34.41
Disappointment	94	23.27
Headache	85	21.04
Nausea	69	17.08
Depression	67	16.58
Vomiting	34	8.41

Related with symptoms, 180 (44.55%) students passed the time without any problem. Others 224(55.45%) students faced different problems. From them 126 (56.25%) had problem of attention in the class (Table 4).

**Table 4:** Problems face during menses among undergraduate students of DBU, Ethiopia participated in the study 2020 (N = 224).

Problem	Categories	Number	Percent (%)
No attention in the class	Yes	126	56.25
	No	98	43.75
Absent from class	Yes	71	31.69
	No	153	68.30
Decrease class activity	Yes	75	33.48
	No	149	66.52
Low grade achievement	Yes	104	46.43
	No	120	53.57
Absent from exam	Yes	12	5.36
	No	212	94.64

### Effect of menstrual irregularity on academic performance

The mean average grade point (AGP) of study participants was 2.88 with (SD ±0.57). Furthermore, 45% of students had an average grade point above the mean. Students who had menstrual irregularity had mean AGP of (2.78 ± 0.57) and students who had regular menstruation had mean AGP of (2.97 ± 0.53) (Table 5).

**Table 5:** Effect of menstrual irregularity on academic performance of undergraduate students of DBU, Ethiopia 2020.

Variable		Frequency	Mean AGP	Mean difference	95%CI of mean AGP	𝑝 value	Std. Deviation	Mean+Std
Menstruation	Regular	202	2.97	0.19	(0.08–0.30)	0.001	0.53	2.97+0.57
	Irregular	202	2.78				0.57	2.78+0.57

This study found that students who had menstrual irregularity had significantly lower mean average grade point by a mean difference of 0.19 (95%CI: 0.08–0.30) as compared with students who had regular menstruation (Table 5).

## Discussion

The implications of reproductive health on quality of life and other activities are many. This study examines the association between menstrual cycle and academic performance among undergraduate students of Debre Berhan University. In the present study 56.25% of students had no attention in the class, 31.69% were absent from class, 33.48% decrease class activity, 46.43% had low grade achievement and 5.36% were absent from exam during menses. Moreover, students which had menstrual irregularity had significantly lower mean average grade result as compared with students who had regular menstruation.

This is in accordance to the Arabian study, which showed that academic performance was affected by menstruation in several ways, mainly study time (76%), concentration (65.8%), participation in group activities (58.1%), examination performance (51.8%) and class attendance (40.8%) [12].

Another study conducted in Mansoura University revealed that 79.5% of nursing students had sleeping desire during lectures, 78.7% experienced restrictions in practical performance, 77.5% had difficulty in concentration and understanding and 75.1%, decreased participation in discussion. In addition study conducted in Kuwait study 75.5% of the participants perceived menstrual discomfort as having negative effects on their participation [13]. A study conducted on the effects of the different phases of the menstrual cycle on physical working capacity showed that the female resting heart and respiratory rates were decease during the menstrual cycle which have adverse effects on physical work output and sport participation [14].

Our study has limitations to consider when interpreting results. First most data on the participant’s questioner were self-reported even Average grade result so social desirability bias may be present.

## Conclusion

It can be concluded that menstrual irregularity has a major impact on students ‘academic performance. This study found that students who had menstrual irregularity had significantly lower mean average grade point as compared with students who had regular menstruation. But this study give insight about the effect of menstrual irregularity on academic performance, further research should be conducted to study the effect of menstrual irregularity on academic performance on university students.

## Supporting information

S1 Data Data collected data from Debre Berhan university undergraduate students to assess the effect of menstrual irregularity on academic performance 2020. (SAV)

S1 File Questioner used to assess the effect of menstrual irregularity on academic performance 2020. (DOCX)

## Abbreviations

AGP: Average Grade Point
DBU: Debre Berhan University
IFGO: International Federation of Gynecologists and Obstetrics
