# Supplementary material for: Effect of menstrual irregularity on academic performance of undergraduate students of Debre Berhan University: A comparative cross sectional study
Source: PLoS One. 2023 Jan 26;18(1):e0280356. doi: 10.1371/journal.pone.0280356 (PMC9879450; doi:10.1371/journal.pone.0280356)
Supplement: S1 File — (DOCX) [file pone.0280356.s002.docx]

**Questioner**

## English version

Consent form

I am Enguday Demeke, student of Epidemiology in Debre Birhan University. I’m doing research on magnitude and associated factors of menstrual irregularity and its effect on academic performance among undergraduate students of DBU. I kindly invite you to be part of this research. Before you decide, you can talk to anyone you feel comfortable with about the research. I will not be collecting or retaining any information about your identity. Your decision will not result in any loss or benefits to which you are otherwise entitled. You have the right not to answer any single question, as well as to withdraw completely from the interview at any point during the process; additionally, you have the right to request the researcher not to use any of your responses. Your signature below indicates that you have decided to volunteer as a research participant for this study, and that you have read and understood the information provided above.

Subject's Signature: ___________________ Date _____________________

Investigator’s Signature: _______________________________

**Part I- Socio-demographic characteristics**

| S.no | Questions | Response | Remarks |
| --- | --- | --- | --- |
| Q1 | How old are you? | ……years |  |
| Q2 | What is your ethnicity? | 1.Amhara □ 2. Oromo □ 3.Tigrie □ 4 Others (specify)… |  |
| Q3 | What is your religion? | 1.Orthodox □ 2.Protestant □  3.Muslim □ 4. Others (specify) --- |  |
| Q4 | Where is your permanent resident address before coming to this university? | 1.Urban □ 2.Rural □ |  |
| Q5 | What is your marital status? | 1.Single□ 2.Married□ 3.Divorced □ 4. Widowed |  |
| Q6 | Birr sent from family per month? | …..Birr |  |
| Q7 | Your academic year (Bach)? | 1.1^st^ year □ 2. 2^nd^ year □ 3. 3^rd^ year □ 4.4^th^ year □ 5.5^th^ year □ |  |

| **Part II- Menstrual cycle pattern** | | | |
| --- | --- | --- | --- |
| S.no | Questions | Response | Remarks |
| Q8 | How old were you when you start menstruating? | ____years |  |
| Q9 | Did your period return on the same interval/day? | 1.Yes□ 2.No □ | If yes skip to q12 |
| Q10 | If no to q9 how long was the longest period in the last one year? | ____Days |  |
| Q11 | If no to q9, how long was the shortest period in the last one year? | ____days |  |
| Q12 | If yes to q9, how long was the interval between the first day of one menses and the first days of next menses? | ….days |  |
| Q13 | How many days does your menses last? | ____days |  |
| Q14 | What was you used for menstrual hygiene management? | 1.Cloth/Towel□ 2.Modes □  3.Others (specify) __ |  |
| Q15 | The number of Cloth/modes used in a day during the heaviest day of menses? | ….. |  |
| Q16 | Can you determine your amount of bleeding? | 1.Light□ 2.Normal□ 3.Heavy □ |  |
| Q17 | What type of symptom you face during menstruation? you can choose more than one | 1. no symptom  2.Headache□3.AbdominalCramp□4.Backpain□5.Vomiting□6.Nausea□7.Disappointment□ 8Depression 9.Others (specify)… |  |
| Q18 | What treatment you receive during your menstrual pain? | 1.Rest□2.drug□ 3.Coffee/tea□ 4.Others (specify)… | If not 2 skip toq20 |
| Q19 | If drug for q18 did the pain relive by the drug? | 1.yes 2.no |  |
| Q20 | Which problems you face during menses? | 1.No problem □ 2.Absent from class □ 3.No attention in the class □ 3.Decrease class activity □ 4.Absentfrom examination□5. Low grade achievement□6. Others specify…… |  |
| Q21 | Put your average grade result of the last semester | …. |  |

**Medical and medication history**

| S.no | Questions | Response | Remarks |
| --- | --- | --- | --- |
| Q22 | Do you have history of diagnosed thyroid disorders? | 1.Yes □ 2.No □ |  |
| Q23 | Do you have history of diagnosed sexually transmitted disease or reproductive tract infection? | 1.Yes □ 2.No □ |  |
| Q24 | Do you have history of diagnosed diabetes mellitus? | 1.Yes □ 2.No □ |  |
| Q25 | Do you have history of diagnosed hypertension? | 1.Yes □ 2.No □ |  |
| Q26 | Do you have history of diagnosed anemia? | 1.Yes □ 2.No □ |  |
| Q27 | Are you HIV/AIDS positive? | 1.Yes □ 2.No □ |  |
| Q28 | Do you have history of head injury | 1.Yes □ 2.No □ |  |
| Q29 | Have you started sexual intercourse? | 1.Yes □ 2.No □ |  |
| Q20 | Do you take any form of contraceptives? | 1.Yes □ 2.No □ | If no skip to q31 |
| Q31 | If yes to q29, what form of contraceptive? | 1.Pills □ 2.Injectable □ 3.Norplant□ others(Specify)___ |  |
| Q32 | Do you take any antipsychotic drugs? | 1.Yes □ 2.No □ |  |

**Life style and behavioral factors**

| S.no | Questions | Response | Remarks |
| --- | --- | --- | --- |
| Q33 | Have you ever smoke cigarrate in your life? | 1.Yes□ 2.No□ | If no skip to q35 |
| Q34 | If yes to q32 do you smoke currently? | 1.Yes□ 2.No □ | If no skip to q35 |
| Q35 | If yes to q9 number of cigarettes you smoke? | 1…..daily 2…weekly |  |
| Q36 | How many hours do you sleep on average per day | ….hours |  |
| Q37 | How often do you have a drink containing alcohol? | 1. Never□ 2. Monthly or less□ 3. 2-4 times a month □ 4. 2-3 times a week□ 5. >=4 times per week□ |  |
| Q38 | How many drinks containing alcohol do you have on atypical day when you are drinking? | 1.1- 2 □ 2. 3 - 4□ 3. 5 - 6 □ 4. 7-9□ 5.>=10□ |  |
| Q39 | How often do you have six or more drinks in one occasion? | 1.never□ 2.less than monthly□ 3.monthly□ 4.2-3 times/week□ 5.>=4 times/week□ |  |
| Q40 | How often during the last year have you found that you were not able to stop drinking once you have started? | 1.never□ 2.less than monthly□ 3.monthly□ 4.2-3 times/week□ 5.>=4 times/week□ |  |
| Q41 | How often during the last year have you failed to do which was normally expected from you because of drinking | 1.never□ 2.less than monthly□ 3.monthly□ 4.2-3 times/week□ 5.>=4 times/week□ |  |
| Q42 | How often during the last year have you have you needed a first drink in the morning to get yourself going after a heavy drinking session? | 1.never□ 2.less than monthly□ 3.monthly□ 4.2-3 times/week□ 5.>=4 times/week□ |  |
| Q43 | How often during the last year have you had a feeling of guilt/remorse after drinking? | 1.never□ 2.less than monthly□ 3.monthly□ 4.2-3 times/week□ 5.>=4 times/week□ |  |
| Q44 | How often during the last year have you been unable to remember what happed the night before b/c you have been drinking? | 1.never□ 2.less than monthly□ 3.monthly□ 4.2-3 times/week□ 5.>=4 times/week□ |  |
| Q45 | Have you or someone else been injured as a result of your drinking? | 1.no□ 2.yes but not in the last year□  3. yes during the last year□ |  |
| Q46 | Has a relative/friend/doctor been concerned about your drinking? | 1.no□ 2.yes but not in the last year□  3. yes during the last year□ |  |

| **Physical activity** |
| --- |
| Q47. During the last 7 days, did you do vigorous physical activities that cause large increases in breathing or heart rate like aerobics, running, weight lifting, Gymnastic or fast bicycling? ( Think about only those physical activities that you did for at least 10 minutes at a time)  Yes □ No □ if no skip to q50  Q48. If yes for Q47 how many days per week? ________________  Q49. How much time in total did you usually spend on one of those days doing vigorous physical activities? _____ hours ______ minutes  Q50. During the last 7 days, did you do moderate physical activities that cause a small increase in breathing or heart rate like carrying light loads, bicycling at a regular pace, swimming, volleyball? Do not include walking.( Think about only those physical activities that you did for at least 10 minutes at a time)  Yes □ No □ if no skip to q53  Q51. If yes for Q50 how many days per week? ________________  Q52. How much time in total did you usually spend on one of those days doing moderate physical activities? _____ hours ______ minutes  Q53. During the last 7 days, on how many days did you walk for at least 10 minutes at a time? This includes walking to travel from place to place, and any other walking that you did solely for recreation, sport, exercise or leisure.  Q54 How many days per week? ________________  Q55. How much time in total did you usually spend on one of those days doing moderate physical activities? _____ hours ______ minutes |

**Stress factor**

| S.no | Questions | Response | Remarks |
| --- | --- | --- | --- |
| Q56 | In the last month, how often have you been upset because of something that happened unexpectedly? | 1.Never □ 2.Sometimes□ 3.Very often□ |  |
| Q57 | In the last month, how often have you felt that you were unable to control the important things in your life? | 1.Never□ 2.Sometimes□ 3.Very often□ |  |
| Q58 | In the last month, how often have you felt nervous and “stressed”? | 1.Never □ 2.Sometimes□  3.Very often□ |  |
| Q59 | In the last month, how often have you felt confident about your ability to handle your personal problems? | 1.Never □ 2.Sometimes□  3.Very often□ |  |
| Q60 | In the last month, how often have you felt that things were going your way? | 1.Never □ 2.Sometimes□  3.Very often□ |  |
| Q61 | In the last month, how often have you been able to control irritations in your life? | 1.Never □ 2.Sometimes□  3.Very often□ |  |
| Q62 | In the last month, how often have you been angered because of things that were outside of your control? | 1.Never □ 2.Sometimes□  3.Very often□ |  |

**Part III Anthropometric measurements of the participant**

Weight (Kg) ---------- Height (cm) -------- BMI-----

**Part IV Physical examination** Thyroid examination…

Inspection 1. Not visible 2. Visible in normal position

Palpation 1. Palpable 2. Not palpable

## Amharic version

**መረጃ ለመስጠት ፈቃደኛ መሆንሽን ማረጋገጫ**

እኔ ስሜ እንጉዳይ ደመቀ ሲባል በደብረብረሃን ዩኒቨርሲቲ በህብረተሰብ ጤና የሁለተኛ ዲግሪየን በመማር ላይ እገኛለሁ፡፡ በመሆኑም የዲግሪ ማሙያ ጥናቴን በወር አበባ መዛባተና መንስዒው ላይ እየሰራሁ ስለሆነ በዚህ ጥናት ዉስጥ እንዲሳተፉ ጥያቄ ላቀርብልዎት እወዳለሁ፡፡ በጥናቱ ለመሳተፍ ከመወሰንዎ በፊት የሚያሳስብዎት ነገር ካለ ከዋናዉ ተመራማሪ እና ከፈለጉት አካል ጋር የመነጋገር ሙሉ ነጻነት አለዎት፡፡ በጥናቱ ስዓት ማንነትዎን የሚገልጽ ምንም አይነት መረጃ የማንዎስድ ሲሆን የሚሰጡት መረጃ ሙሉ በሙሉ በሚስጥር የሚያዝ ይሆናል፡፡ በዚህ ጥናት በመሳተፍዎ የሚደርስብዎት ምንም አይነት ጉዳት የሌለ ሲሆን የሚያገኙትም ቀጥተኛ ጥቅም አይኖርም፤ ከዛ ባሻገር ግን ችግሩ ያለበትን ደረጃ ለማወቅ የድርሻዎን ይወጣሉ፡፡ የፈለጉትን ጥያቄ ያለመመለስ የመለሱትን ጥያቄ ተመራማሪዉ እንዳይጠቀም የመጠየቅ መብትዎ የተጠበቀ ነዉ፡፡ ይህንን ተከትሎ ከታች የሚፈርሙት ፊርማ በዚህ ጥናት ለመሳትፍ ፈቃደኛ መሆንዎንና ከላይ የተሰጡትን መረጃወች አንብበዉ የተረዱ መሆኑን ያረጋግጣል፡፡

ፊርማ____________________________

ቀን____________________________

| **ክፍል አንድ፡- ማህበራዊ እና ስነ ህዝብ መረጃ መጠይቅ** |
| --- |
| 1. እድሜ ？**____________________** ዓመት |
| 1. ብሄር 1. አማራ 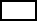 2. ኦሮሞ 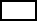 3. ትግሬ 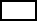 ሌላ ከሆነ**：**———————ይጥቀሱ |
| 1. ሐይማኖት？ 1. ኦርቶዶክስ 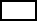 2.ፕሮቴስታንት 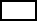 3.ሙስሊም 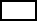 4. ሌላ ከሆነ**：**———————ይጥቀሱ |
| 1. የመጡበት ቦታ？ 1.ከተማ 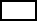 2. ከፍለ ሃገር 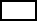 |
| 1. የጋብቻ ሁኔታ？ 1.ያላገባ 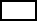 2.ያገባ 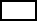 3.የተፋታ 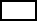 4.የሞተበት 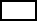 |
| 1. በወር ከቤተሰብ የሚላክልወት ብር ？**________**ብር |
| 1. የትምህርት አመት？ 1.አንደኛ አመት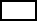 2.ሁለተኛ አመት 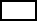 3.ሶስተኛ አመት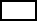 4.አራተኛ አመት 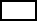 5.አምስተኛ አመት 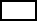 |
| 1. የወር አበባ ማየት የጀምሩት በስንት አመትወ ነበር？**_____** አመት |
| 1. የወር አበባወ በየወሩ በተመሳሳይ ቀን ይመጣል？1.አወ 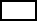 2.አይመጣም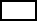 (መልስዎ አወ ከሆነ ወደ ቁጥር 1**2** ይለፉ) |
| 1. መልስዎ አይመጣም ከሆነ ባለፈው አመት ዉስጥ የወር አበባይ ዘገየብኝ ያሉት በስንት ቀኑ ሲመጣ ነዉ？ **_____**ቀን |
| 1. መልስዎ አይመጣም ከሆነ ባለፈው አመት ዉስጥ የወር አበባይ ተሎ መጣ ያሉት በስንት ቀኑ ሲመጣ ነዉ？ **_____**ቀን |
| 1. ለጥያቄ ቁጥር 9 መልስዎ አወ ከሆነ የወር አበባወ በአማካይ በየስንት ቀኑ ይመጣል ？ **_____**ቀን |
| 1. የወር አበባወ ከመጣበት እስከሚጠራበት ድረስ ለስንት ቀናት እየፈሰሰ ይቆያል? **____**ቀን |
| 1. በወር አበባወ ጊዜ የሚጠቀሙት ምንድን ነበር**？** 1.ፎጣ 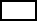 2.ሞደስ 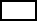 ሌላ ከሆነ **：**————— ይጥቀሱ |
| 1. በጣም በሚበዛበት ጊዜ በቀን ሰንት ሞደስ ወይም ፎጣ ይጠቀማሉ**？___** |
| 1. በራሰዎ አስተሳሰብ የወር አበባወን የፍሰት መጠን ምን ይላሉ **？**1.ቀላል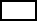 **2.**ትክክለኛ 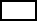 3.ብዙ 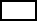 |
| 1. የወር አበባወ በሚመጣበት ጊዜ የሚሰማወት ስሚት የትኛው ነው**？**(ከአንድ በላይ መምረጥ ይችላሉ) 1. .ችግር የለም 2.የራስ ህመም 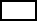 3.የሆድ ቁርጠት 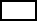 4.የጀርባ ህመም 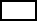 5.ማስታወከ 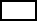 6.ማቅለሽለሽ 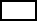 7.መበሳጨት 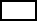 8. ጭንቀት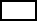 8.ሌላ ካለ **：**—— ይጥቀሱ |
| 1. የወር አበባ ህመም በሚሰማዎት ጊዜ ምን ይጠቀማሉ**？** 1.እረፍት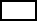 . 2.ማሰታገሻ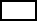 3.ሻይ ወይም ቡና 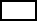 4.ሌላ ካለ **：**—— ይጥቀሱ( ለጥያቄ ቁጥር 18 ማሰታገሻ ካላሉ ወደ ጥያቄ 20 ይለፉ) |
| 1. ለጥያቄ ቁጥር 18 ማሰታገሻ ካሉ ምደሃኒቱ ህመምወን ያስታግስለወታል? 1. አዎ 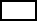 2. አያስታግስልኝም 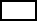 |
| 1. የወር አበባወ በሚመጣበት ጊዜ የትኞቹ ችግሮች ያጋጥምዎታል? 1.ችግር የለም 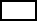 2. ከክፍል መቅረት 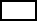 3. በክፍል ውስጥ ትኩረት ማጣት 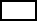 3.የክፍል ሥራ እንቅስቃሴ መቀነሰ 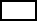 4ከፈተና መቅረት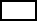 5. ዝቅተኛ ውጤት ማምጣት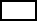 . 6. ሌሎችን ይጥቀሱ …… |
| 1. የመጨረሻውን ሴሚስተር ውጤትዎን ያስቀምጡ………. |
| 1. በጤና ባለሙያ የታይሮይድ ዕጢ በሽታ አለብዎት ተብለው ያውቃሉ? 1.አዎ 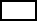 2. አላውቅም 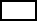 |
| 1. በጤና ባለሙያ በግብረ-ሥጋ ግንኙነት የሚተላለፉ በሽታዎች ወይም የመራቢያ አካላት በሽታዎች አለብዎት ተብለው ያውቃሉ? 1.አዎ 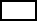 2. አላውቅም 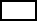 |
| 1. በጤና ባለሙያ የስኳር በሽታ አለብዎት ተብለው ያውቃሉ? 1.አዎ 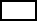 2. አላውቅም 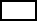 |
| 1. በጤና ባለሙያ የደም ግፊት አለብዎት ተብለው ያውቃሉ? 1.አዎ 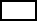 2. አላውቅም 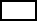 |
| 1. በጤና ባለሙያ የደም ማነስ አለብዎት ተብለው ያውቃሉ? 1.አዎ 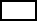 2. አላውቅም 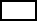 |
| 1. በጤና ባለሙያ የኤችአይቪ / ኤድስ ቫይረስ አለብዎት ተብለው ያውቃሉ? 1.አዎ 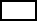 2. አላውቅም 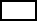 |
| 1. የጭንቅላት ጉዳት ታሪክ አለዎት? 1.አዎ 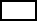 2. የለም 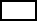 |
| 1. የግብረ ሥጋ ግንኙነት ጀምረዋል? 1.አዎ 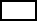 2. አልጀመርኩም 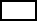 |
| 1. የወሊድ መከላከያ ይወስዳሉ? 1.አዎ 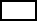 2አልወስድም. 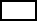 (መልሰው አልወሰድም ከሆነ ወደ ጥያቄ 32 ይለፉ) |
| 1. ለጥያቄ 30 መልስዎ አዎ ከሆነ ምን ዓይነት የእርግዝና መከላከያ ዘዴ ተጠቀሙ? 1.የሚዋጥ 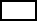 2.በመርፊ የሚሰጥ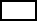3.በከንድ የሚቀበር 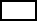 4..ሌላ ከሆነ **：**—— ይጥቀሱ |
| 1. የአእምሮ በሽታ መድኃኒቶችን ይወስዳሉ? 1.አዎ 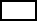 2. አልወስድም 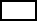 |
| **የስነምግባር** **ጥያቄዎች** |
| 1. በሕይወትዎ ውስጥ ሲጋራ አጭሰዉ ያውቃሉ? 1.አዎ 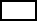 2. አላውቅም 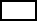 መልሰው አላውቅም ከሆነ ወደ ጥያቄ 36 ይለፉ |
| 1. ለጥያቄ 33 መልሰው አዎ ከሆነ አሁንም ያጨሳሉ? 1.አዎ 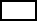 2. አላጨስም 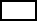 መልሰው አላጨስም ከሆነ ወደ ጥያቄ 36 ይለፉ |
| 1. ለጥያቄ 34 መልሰው አዎ ከሆነ ስንት ሲጋራ ያጨሳሉ? በቀን………. በሳምንት…………. |
| 1. በየቀኑ ባማካኝ ስንት ሰዓት ያተኛሉ? ……..ስአት |
| 1. አልኮሆል ያለው መጠጥ ምን ያህል ጊዜ ይጠጣሉ? 1. በጭራሽ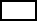 2. በወር ወይም ከዚያ በታች 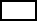 3. በወር 2-4 ጊዜ 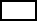   4. በሳምንት 2-3 ጊዜ 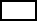 5. በሳምንት 4 ጊዜ እና ከዚያ በላይ 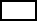 |
| 1. በሚጠጡበት ጊዜ ስንት መጠጥዎችን ይጠጣሉ? 1.1- 2 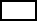 2. 3 – 4 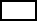 3. 5 - 6 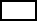 4. 7-9 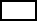 5.>=10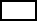 |
| 1. በአንድ ጊዜ ውስጥ ምን ያህል ጊዜ ስድስት ወይም ከዚያ በላይ መጠጦች ይጠጣሉ? 1. በጭራሽ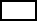 2. በወር ወይም ከዚያ በታች 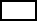 3. በወር 1 ጊዜ 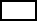 4. በሳምንት 2-3 ጊዜ 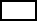 5. በሳምንት 4 ጊዜ እና ከዚያ በላይ 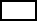 |
| 1. ባለፈው ዓመት ውስጥ ምን ያህል ጊዜ መጠጣት ከጀመሩ በኋላ ማቆም እንዳልቻሉ አገኙት ? 1. በጭራሽ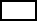 2. በወር ወይም ከዚያ በታች 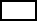 3. በወር 1-2 ጊዜ 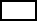 4. በሳምንት 2-3 ጊዜ 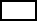 5. በሳምንት 4 ጊዜ እና ከዚያ በላይ 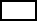 |
| 1. ባለፈው ዓመት ውስጥ በመጠጥዎ ምክንያት ምን ያህል ጊዜ በመደበኛነት ከእርስዎ የሚጠበቀውን ስራ ሳይሰሩ ቀሩ ? 1. በጭራሽ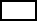 2. በወር ወይም ከዚያ በታች 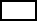 3. በወር 1-2 ጊዜ 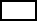 4. በሳምንት 2-3 ጊዜ 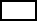 5. በሳምንት 4 ጊዜ እና ከዚያ በላይ 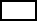 |
| 1. ባለፈው ዓመት ውስጥ ለምን ያህል ጊዜ ከከባድ መጠጥ በኋላ ጠዋት ለመሄድ መጠጥ ፈለጉ? 1. በጭራሽ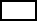 2. በወር ወይም ከዚያ በታች 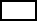 3. በወር 1-2 ጊዜ 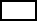 4. በሳምንት 2-3 ጊዜ 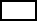 5. በሳምንት 4 ጊዜ እና ከዚያ በላይ 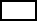 |
| 1. ባለፈው ዓመት ውስጥ ከጠጡ በኋላ ለምን ያህል ጊዜ የጥፋተኝነት / የመጸፀት ስሜት ተሰምቶዎት ነበር?   1. በጭራሽ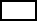 2. በወር ወይም ከዚያ በታች 3. በወር 1-2 ጊዜ 4. በሳምንት 2-3 ጊዜ 5. በሳምንት 4 ጊዜ እና ከዚያ በላይ |
| 1. ባለፈው ዓመት ውስጥ ለምን ያህል ጊዜ በመጠጥዎ ምክንያት ምሽት ምን እንደተፈጠረ ማስታወስ አልቻሉም?1. በጭራሽ 2. በወር ወይም ከዚያ በታች 3. በወር 1-2 ጊዜ 4. በሳምንት 2-3 ጊዜ 5. በሳምንት 4 ጊዜ እና ከዚያ በላይ |
| 1. በመጠጥዎ ምክንያት እርስዎ ወይም ሌላ ሰው የቆስለ ነበር? 1. የለም 2. አወ ግን ባለፈው ዓመት አይደለም  3. አዎ ባለፈው ዓመት |
| 1. የእርስዎ አልኮል መጠጣት ለዘመድ / ጓደኛ / ዶክተር አሳስቦት ነበር? 1. የለም 2. አወ ግን ባለፈው ዓመት አይደለም  3. አዎ ባለፈው ዓመት |
| **የአካል ብቃት እንቅስቃሴ ጥያቄዎች** |
| 1. በአለፉት 7 ቀናት ውስጥ እንደ መሮጥ ፣ ክብደት ማንሳት ፣ ጂምናስቲክስ ወይም ፈጣን ብስክሌት መንዳት ያሉ ከፍተኛ የመተንፈስ ወይም የልብ ምት መጨመር የሚያስከትሉ ጠንካራ የአካል ብቃት እንቅስቃሴዎችን አድርገዋል? (በአንድ ጊዜ ቢያንስ ለ 10 ደቂቃ ያከናወናቸውን አካላዊ እንቅስቃሴዎችን ብቻ ያስቡ)   1.ሰርቻለሁ 2. አልሰራሁም (መልስዎ አልሰራሁም ከሆነ ወደ ጥያቄ ቁጥር 50 ይለፉ) |
| 1. ከሰራህሽ በሳምንት ለስንት ቀን ____________________________________ |
| 1. ከሰራህሽ በቀን ለምን ያህል _____ ሰዓታት ______ ደቂቃዎች |
| 1. በአለፉት 7 ቀናት ውስጥ እንደ ቀላል ጭነት ፣ በመደበኛ ፍጥነት ብስክሌት መንዳት ፣ መዋኘት ፣ ኳስ መ ጫወት የመሳሰሉ ትንፋሽ ወይም የልብ ምት እንዲጨምር የሚያደርግ መጠነኛ የአካል ብቃት እንቅስቃሴ ሰርተዋል ? (መራመድን) አያካቱ (ቢያንስ ለ 10 ደቂቃዎች በአንድ ጊዜ ስላከናወኗቸው አካላዊ እንቅስቃሴዎች ብቻ ያስቡ) 1.ሰርቻለሁ 2. አልሰራሁም (መልስዎ አልሰራሁም ከሆነ ወደ ጥያቄ ቁጥር 53 ይለፉ) |
| 1. ከሰራህሽ በሳምንት ለስንት ቀን________________ |
| 1. ከሰራህሽ በቀን ለምን ያህል _____ ሰዓታት ______ ደቂቃዎች |
| 1. በአለፉት 7 ቀናት ውስጥ ቢያንስ ለ 10 ደቂቃዎች በእግር ሂደዉ ነበር? ይህ ከቦታ ወደ ቦታ መጓዝን ፣ለስፖርት ወይም ለመዝናኛ ያከናወናቸውን ማናቸውንም መጓዝን ያካትታል ) 2. አወ 2. አልሄድኩም (መልስዎ አልሄድኩም ከሆነ ወደ ጥያቄ ቁጥር 56 ይለፉ) |
| 1. በሳምንት ስንት ቀናት? ________________ |
| 1. በቀን ለምን ያህል _____ ሰዓታት ______ ደቂቃዎች |
| **የጭንቀት ሁኔታ ጥያቄዎች** |
| 1. ባለፈው ወር ባልተጠበቀ ነገር የተነሳ ምን ያህል ጊዜ ተቆጥተው ነበር? 1. በፍፁም 2. አንዳንድ ጊዜ 3.ሁልጊዜ |
| 1. በአለፈው ወር በሕይወትዎ ውስጥ አስፈላጊ የሆኑትን ነገሮች ለመቆጣጠር እንዳልቻሉ ስንት ጊዜ ይሰማዎታል? 1. በፍፁም 2. አንዳንድ ጊዜ 3.ሁልጊዜ |
| 1. ባለፈው ወር ውስጥ ምን ያህል ጊዜ በጭንቅት ተወጥረው ነበር?1.በፍፁም 2. አንዳንድ ጊዜ 3.ሁልጊዜ |
| 1. ባለፈው ወር ውስጥ የግል ችግሮችዎን በራሰወ ለመፍታት በራሰ የመተማምን ስሚትወ ምን ያህል ነበር ? 1.በፍፁም 2. አንዳንድ ጊዜ 3.ሁልጊዜ |
| 1. ባለፈው ወር ውስጥ ነገሮች አንች በምታስቢው መንገድ ሂደዋል ብለሽ ታስቢለሽ? 1.በፍፁም 2. አንዳንድ ጊዜ 3.ሁልጊዜ |
| 1. ባለፈው ወር ውስጥ ብስጭቶችን ለመቆጣጠር ምን ያህል ጊዜ ቻሉ? 1.በፍፁም 2.አንዳንድ ጊዜ 3.ሁልጊዜ |
| 1. ባለፈው ወር ከቁጥጥርው ውጭ በሆኑ ነገሮች የተነሳ ምን ያህል ጊዜ ተናደው ነበር? 1.በፍፁም 2.አንዳንድ ጊዜ 3.ሁልጊዜ |

ክፍል III አንትሮቦሜትሪ መለኪያዎች እና የተሳታፊውን አካላዊ ምርመራ

ክብደት (ኪ.ግ.) ---------- ቁመት (ሴ.ሜ) -------- የታይሮይድምርመራ በማየት .......... በመዳሰስ ..........
